# Supplementary material for: Home sick: impacts of migratory beekeeping on honey bee (Apis mellifera) pests, pathogens, and colony size
Source: PeerJ. 2018 Nov 2;6:e5812. doi: 10.7717/peerj.5812 (PMC6216951; doi:10.7717/peerj.5812)
Supplement: Table S2 — Primers used for the amplification of RNA viral amplicons and Apis mellifera actin gene. [file peerj-06-5812-s002.pdf]

Primer	5' to 3' Sequence	Product Size (bp)	Annealing Temp (^{o}C)	Reference
DWV-F	TTCATTAAAGCCACCTGGAACATC	136	53	Traynor et al., 2016
DWV-R	TTTCCTCATTAACTGTGTCGTTGA			
BQCV-F	TTTAGAGCGAATTCGGAAACA	140	51	Traynor et al., 2016
BQCV-R	GGCGTACCGATAAAGATGGA			
IAPV-F	CCATGCCTGGCGATTCAC	203	47	Traynor et al., 2016
IAPV-R	CTGAATAATACTGTGCGTATC			
Actin-F	CGTGCCGATAGTATTCTTGC	138	56	
Actin-R	CCATTGTCAACTACGAGTGC			
