# Supplementary material for: Home sick: impacts of migratory beekeeping on honey bee (Apis mellifera) pests, pathogens, and colony size
Source: PeerJ. 2018 Nov 2;6:e5812. doi: 10.7717/peerj.5812 (PMC6216951; doi:10.7717/peerj.5812)
Supplement: Data S1 — Entire sequence used to develop gBlocks Gene Fragments (Integrated DNA Technologies). Each target of interest in color coded for visualization. green, DWV, blue, IAPV, red, Actin, yellow, IAPV. Ten random base pairs (uncolored) flank each target of interest. [file peerj-06-5812-s004.pdf]

GGACGGACAGTTCATTAAAGCCACCTGGAACATCAGGTAAGCGATGGTTGTT
TGACATTGAGCTACAAGACTCGGGATGTTATCTCTTGCGTGGAATGCGTCCCG
AACTTGAGATTCAATTATCAACGACACAGTTAATGAGGAAAACCATGTACGC
CATGCCTGGCGATTCACAACAAGAAAGCAATACTCCCAATGTACACAACACG
GAACTCGCTTCGTCAACTAGTGAAAACTCGGTTGAGACCCAAGAAATCACAA
CCTTTCATGATGTGGAAACTCCAAATAGGATCGATACCCCCATGGCTCAGGA
TACTTCATCGGCTAGGAACATGGATGATACGCACAGTATTATTCAGCTTCCCT
GCTCGTGCCGATAGTATTCTTGCGGTGTCTCTTTGCCGATCAACGATCGTGTA
CTTTGTTGGTTACCTTCGATTCTAAAAGATAACTCAATAAACCAAACATGTGT
GACGAAGAAGTTGCTGCACTCGTAGTTGACAATGGCGTCCACCTGTTTAGAG
CGAATTCGGAAACATTTTACTATAGTTCAGGTCGGAATAATCTCGATATAGCC
ACTTCACCTCCTTCCATCAATCGCTACTATGCGGTAGGTGCGGGAGATGATAT
GGACTTTTCCATCTTTATCGGTACGCC ATGAGCGCCA
