# Supplementary figures and images for: Home sick: impacts of migratory beekeeping on honey bee (Apis mellifera) pests, pathogens, and colony size
Source: PeerJ. 2018 Nov 2;6:e5812. doi: 10.7717/peerj.5812 (PMC6216951; doi:10.7717/peerj.5812)

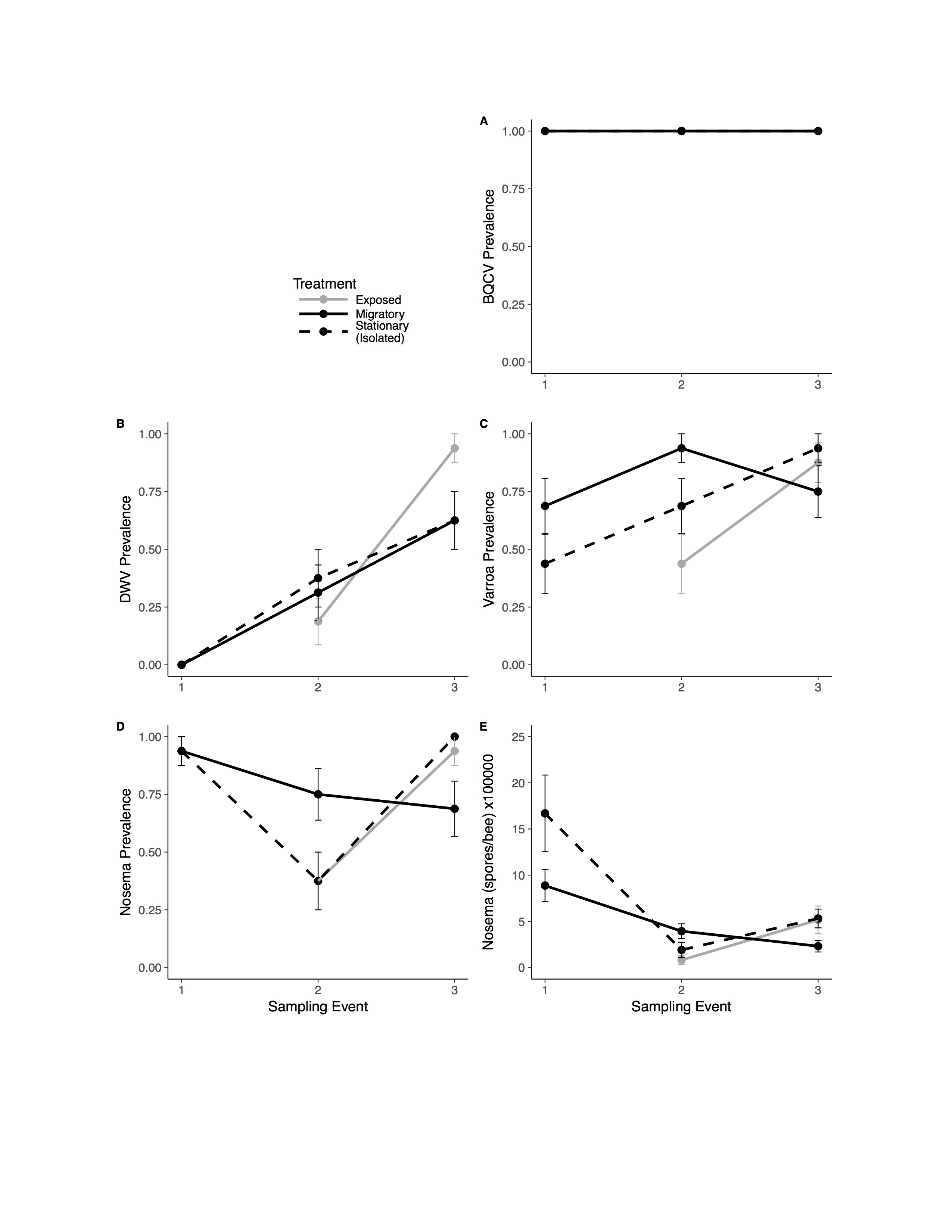

Supplement: Figure S1 — Migratory (solid line) and Stationary/Isolated (dotted line) colonies were sampled at three time points and Exposed (gray) colonies were sampled at two time points. Sampling event (1) occurred before migratory colonies were transported, (2) upon their return, and (3) one month after return. Panels show results for five pathogens and one health metric: (A) black queen cell virus (BQCV) prevalence (B) deformed wing virus (DWV) prevalence (C) Varroa prevalence (D) Nosema prevalence (E) Nosema load (spores per bee) times 100,000. [file peerj-06-5812-s005.jpg]
